# Smithsonian Contributions to Knowledge

**Published:** 1857-09

**Authors:** 


ART. VI.—*Smithsonian Contributions to Knowledge. Investigations, Chemical and Physiological, relative to certain American Vertebrata.* By JOSEPH JONES, M.D., Professor of Chemistry in the Savannah Medical College, 1856.

WE address ourselves, with extreme regret, to the duty of briefly reviewing the memoir of Professor Jones. We say, with extreme regret, because it is never pleasant to expose the defects of an author, and because in the present case, the defects are almost atoned for by an industry and perseverance, which in time will, no doubt, attain to more perfect and more accurate results.

Professor Jones treats, in the book before us, of the method of analyzing blood, of the blood of vertebrated animals in normal and abnormal conditions, of the effects of starvation on their solids and fluids, of digestion, of the comparative anatomy and physiology of the pancreas, liver, and spleen, and finally, of the kidney and its secretion.

The range of subjects thus considered is enormous for the scope of a memoir, and we shall, therefore, hasten to pass over the ground with a rapidity proportioned to its extent. The chapter upon Analysis exhibits, at the outset, a defect, or rather an excess, which exists throughout the volume. In place of a simple, definite statement of the author's plan of chemical procedure, we are entertained with an account of how this and that man's method is in fault, &c. In other places, as on page 30, where a theory of Professor Carpenter is stated without acknowledgment, the author indulges his reader with the views of Bernard, Professor Jackson, &c. To such an extent is this sometimes carried, that one is puzzled as to what the writer himself conceived to be original, and what he did not.

The next chapter deals with analyses of the blood of animals in a *normal* condition, as the succeeding one treats of it under abnormal conditions. On a general survey several points arrest our attention. There are twenty analyses of *normal* blood, so called. Now will Professor Jones inform us how the blood can be normal in a dog "poorly fed" (Anal. 19), or was he poorly fed on one occasion only, or how long exactly? Next, in casting our eyes over the analyses, we observe that Professor Jones estimates the water, corpuscles, albumen, fibrine, saline constituents, &c., in each specimen of blood. In each case (we have added up most of them), the analysis counts up exactly 1000. A degree of accuracy most astonishing, when we observe that he estimates the fibrine, by the rod or whipping process, of necessity subjecting his analysis, at least in some cases, to a loss of which we have no record. We observe, again, that where the fibrine is unstable, and redissolves in the blood on standing, it is entirely left out of the calculation, in place of being estimated as dissolved albumen. This appearance of remarkable analytic skill, with evidence here and there of singular want of chemical resources, is not a little difficult to understand. Another example occurs on page 63, where, at the foot of a complex analysis, we are told that the specific gravity of the blood was not taken, because our author's last specific gravity bottle was broken; as if any thin phial would not have answered. In several instances where our author restates his former analyses, they are numbered as if new, an error which would undoubtedly mislead a person who had occasion to quote or mention their whole number. Of minor defects there are many, and some which will disappear no doubt in a future edition. Thus, the appearance in the blood, alluded to on page 32, was probably only the well-known molecular fat so often seen in the blood at the close of digestion, and before the assimilation of the absorbed food is completed. Again, in the upper table of specific gravities, on page 24, our author quotes 1046 as the specific gravity of an alligator's blood, whereas on page 11, this is set down as the specific gravity of its defibrinated blood. He omits in this table the specific gravity of the heron's blood, and if we correct the error, and supply the omission, we shall find that the table does not quite so well support his proposition, that "the blood becomes more concentrated as the organs and apparatus and intelligence of animals are developed." Again, is not our author aware that the pulse of the old is *more* rapid than that of adult life, and not slower, as he states it to be.

Perhaps the best part of the book is the anatomical portion, at the close. Here, however, as elsewhere, we are left to discover how much is new, and how much is old knowledge; and while the facts recorded are not only valuable and interesting, the inferences therefrom, and the strictures upon other observers, are anything but successful or well founded—we refer more especially to the chapter on Digestion.

Here our fault-finding ends. Professor Jones's numerous propositions rest for support upon the accuracy of his chemical processes, and demand more space for reviewal than we can afford. We turn with pleasure to what we believe to be really good in the book. Professor Jones is never more instructive, as well as entertaining, than where he records his observations upon the habits of animals, and the peculiarities of structure which explain some of his results. With such a menagerie as our author can command, he would be able to teach us a great deal of what we may call the manners and customs of animals; a kind of knowledge rarely attainable, and yet essential to a correct appreciation of the mental endowments of the lower classes of created beings.

The observations of our author upon the effects of thirst and starvation, on warm and cold blooded animals, are very interesting, and well repay the reader, although the same ground has been traversed more or less by Chossat and by Edwards. The tables of the relative size of the heart, and of the rapidity of the circulation, &c., also contain new information, and of a kind which is rarely to be procured without the utmost difficulty, and for which we think that our author deserves the thanks of physiologists. Again, the same praise is due to his tables of the length of the intestinal canal of the animals examined; the weights of the pancreas, spleen, &c. Certain of the remarks appended to the chapter upon the spleen, we find extremely interesting in their bearing upon Mr. Gray's theory of the functions of this organ. Finally, whatever else may be said in regard to the book before us, no one can doubt that its author possesses uncommon industry, since, between the 4th of April and the 17th of September, he made numerous dissections and drawings, estimated the weights of organs, and the sp. gr. of blood, and other fluids, and carried on and completed at the same time, forty analyses, and this too, as he himself says, in the heat of a Georgia summer, when he was also obliged to pursue and capture the animals upon which he experimented. Let us hope that the next book from this source may be really and simply "contributions to knowledge," and not to such an extent as this one, a rechauffè of the views of others. The men who read monographs are never tyros in chemistry and physiology, and may be safely supposed to understand well enough the current views in these sciences, as well as the anatomical arrangements of the circulatory organs in the birds, fishes, reptiles, &c.

